# Prognostic value of tumour volume in patients with a poor Karnofsky performance status scale – a bicentric retrospective study

**DOI:** 10.1186/s12883-021-02424-0

**Published:** 2021-11-15

**Authors:** Melanie Barz, Julia Gerhardt, Stefanie Bette, A. Kaywan Aftahy, Thomas Huber, Stephanie E. Combs, Yu-Mi Ryang, Benedikt Wiestler, Marco Skardelly, Irina Gepfner-Tuma, Felix Behling, Friederike Schmidt-Graf, Bernhard Meyer, Jens Gempt

**Affiliations:** 1 Department of Neurosurgery, Technical University Munich, School of Medicine, Klinikum rechts der Isar, Ismaninger Str. 22, 81675 Munich, Germany; 2 Department of Neurosurgery, Helios Klinikum Berlin Buch, Berlin, Germany; 3 Department of Diagnostic and Interventional Radiology, Universitätsklinikum Augsburg, Augsburg, Germany; 4 Department of Neuroradiology, Technical University Munich, School of Medicine, Klinikum rechts der Isar, Munich, Germany; 5 Institute of Clinical Radiology and Nuclear Medicine, University Medical Center Mannheim, Medical Faculty Mannheim, Heidelberg University, Mannheim, Germany; 6 Helmholtz Zentrum Munich (HMGU), Department of Radiation Sciences (DRS), Institute of Innovative Radiotherapy (iRT), Munich, Germany; 7 Deutsches Konsortium für Translationale Krebsforschung (DKTK) (German Cancer Consortium), Partner Site Munich, Munich, Germany; 8 Department of Neurosurgery, University of Tübingen, Tübingen, Germany; 9 Department of Neurology, Technical University Munich, School of Medicine, Klinikum rechts der Isar, Munich, Germany

**Keywords:** Karnofsky performance status scale, Glioblastoma, Surgery, EOR

## Abstract

**Backround:**

Median overall survival (OS) after diagnosis of glioblastoma (GBM) remains 15 months amongst patients receiving aggressive surgical resection, chemotherapy and irradiation. Treatment of patients with a poor preoperative Karnofsky Performance Status Scale (KPSS) is still controversial. Therefore, we retrospectively assessed the outcome after surgical treatment in patients with a KPSS of ≤60%.

**Methods:**

We retrospectively included patients with a de-novo glioblastoma WHO °IV and preoperative KPSS ≤60%, who underwent surgery at two neurosurgical centres between September 2006 and March 2016. We recorded pre- and postoperative tumour volume, pre- and postoperative KPSS, OS, age and MGMT promoter status.

**Results:**

One hundred twenty-three patients (58 females/65 males, mean age 67.4 ± 13.4 years) met the inclusion criteria. Seventy-five of the 123 patients (61%) underwent surgical resection. 48/123 patients (39%) received a biopsy. The median preoperative and postoperative tumour volume of all patients was 33.0 ± 31.3 cm^3^ (IR 15.0–56.5cm^3^) and 3.1 ± 23.8 cm^3^ (IR 0.2–15.0 cm^3^), respectively. The median KPSS was 60% (range 20–60%) preoperatively and 50% (range 0–80%) postoperatively. Patients who received a biopsy showed a median OS for patients who received a biopsy only was 3.0 months (95% CI 2.0–4.0 months), compared to patients who had a resection and had a median OS of 8 months (95% CI 3.1–12.9 months).

Age (*p* < 0.001, HR: 1.045 [95% CI 1.022–1.068]), postoperative tumour volume (*p* = 0.02, HR: 1.016 [95% CI 1.002–1.029]) and MGMT promotor status (*p* = 0.016, HR: 0.473 [95% CI 0.257–0.871]) were statistically significant in multivariate analysis. In subgroup analyses only age was shown as a significant prognostic factor in multivariate analyses for patients receiving surgery (*p* < 0.001, HR: 1.046 [95% CI 1.022–1.072]). In the biopsy group no significant prognostic factors were shown in multivariate analysis.

**Conclusion:**

GBM patients with a preoperative KPSS of ≤60% might profit from surgical reduction of tumour burden.

## Backround

In 1949, Karnofsky and Burchena described their instrument, the Karnofsky Performance Status Scale (KPSS) score, as a numerical scale for quantifying patients’ status in relation to the degree of their independence in daily activities and self-care. Originally, it was used for patients with systemic malignancies and divided them according to their level of activity and medical requirements. Patients are scored into 11 categories from 0 to 100, where, for example, a KPSS of 70% means the patient is able to care for himself but is unable to carry out daily activities [1]. After it had been proven successful in patients with systemic cancer, more and more research groups started to evaluate the KPSS score for brain cancer [2–4]. Previously published studies could show a significant correlation between the preoperative KPSS score and the outcome after glioma surgery [5, 6]. In most studies, only patients suffering from a glioblastoma with a KPSS of ≥70% were included [7, 8]. For example, those studies analysed prognostic factors such as tumour size, GTR and adjuvant therapy modalities postoperatively. However, in our clinical daily work, patients with a noticeably lower KPSS are represented as well. It should be noticed that this can be due to clinic symptomology as seizures, acute mental status changes or focal neurologic deficits caused by tumour size and/or location itself. Therefore, the following study intends to show whether it is worthwhile for patients with a KPSS 60% or below to achieve tumour volume reduction.

## Methods

This retrospective, non-interventional bicentric study was approved by the medical ethics committee of the Technical University Munich (5625–12) and is in accordance with the ethical standards of the 1964 Declaration of Helsinki and its later amendments [9].

### Patient population

**Fig. 1:**
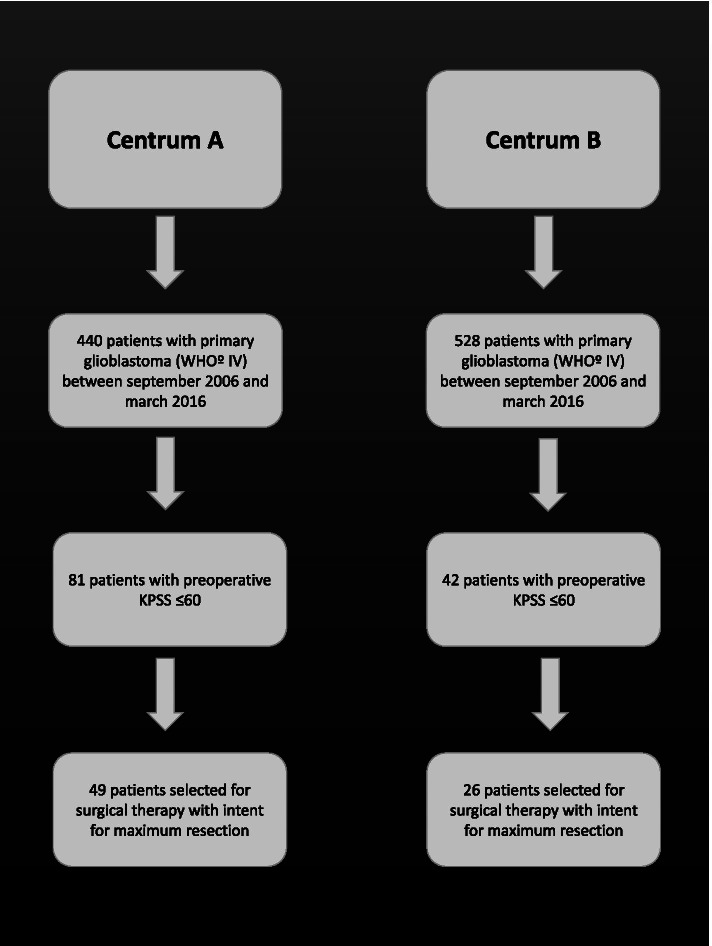
Flowchart of patient-selection process

We retrospectively assessed 968 patients with a histologically confirmed glioblastoma WHO IV with a preoperative Karnofsky Performance Status Scale (KPSS) of ≤60%, who were treated surgically between September 2006 and March 2016 in two neurosurgical departments (Fig. 1). According to interdisciplinary neuro-oncological consensus, patients were assigned to surgery with the intent of complete resection or to biopsy to confirm the histopathological diagnosis.

We retrospectively reviewed pre- and postoperative KPSS, date of initial tumour diagnosis, date of death/last contact, age, sex, adjuvant treatment and histopathological findings from the patients’ medical charts. Also, we performed histopathological analysis according to the WHO criteria of 2016 [10] and quantitatively assessed methylation of the O6-methylguanin-DNA-methyltransferase (MGMT) promoter status. Since the decision regarding adjuvant therapy is made after receipt of the histology in the context of the interdisciplinary neuro-oncological board, depending on the clinical condition, the KPSS was collected approximately 5 days postoperatively.

Then, we calculated the overall survival (OS) from the date of surgery until the date of death or censored for the date of the last patient contact. Only patients with complete magnetic resonance imaging data were included to calculate pre- and postoperative contrast-enhancing tumour volumes. Patients with recurrent tumour or incomplete data were excluded (Table 1).

**Table 1:** Baseline tumour and patient characteristics; normally distributed variables shown as mean ± standard deviation, non-normally distributed as median (interquartile range); KPSS (Karnofsky Performance Status Scale)

Demographic data	
Age	67.4 ± 13.4 years, range 21–90 years
Female	58/123 (47.2%)
Male	65/123 (52.85%)
*Surgical data & tumor burden*	
Biopsy	48/123 (39%)
Median preoperative tumor volume	26.3 ± 30.9 cm^3^ (IR 8.1–51.7 cm^3^)
Median postoperative tumor volume	26.3 ± 30.9 cm^3^ (IR 8.1–51.7 cm^3^)
Resection	75/123 (61%)
Median preoperative tumor volume	35.2 ± 31.3 cm^3^ (IR 19.7–65.3 cm^3^)
Median postoperative tumor volume	0.5 ± 2.8 cm^3^ (IR 0–2.3 cm^3^)
*Karnofsky Performance Status Scale*	
Median preoperative KPSS	60% (20–60%)
Median postoperative KPSS	50% (0–80%)
*Overall survival*	
Median overall survival	5 months (95% CI 3.0–4.0)
Median overall survival after biopsy	3 months (95% CI 2.0–4.0)
Median overall survival after surgery	8 months (95% CI 3.1–12.9)
*MGMT-methylation status*	
MGMT- methylation status available	81/123 (65.9%)
MGMT-methylated	26/81 (32.1%)
MGMT-unmethylated	55/81 (67.9%)

### Imaging

All patients received preoperative and early postoperative MRI (within 72 h after surgery). In centre A, we performed imaging using three different 3 Tesla MRI scanners: Philips Achieva; Philips Ingenia (Philips Medical Systems, The Netherlands B.V.); and Siemens Verio (Siemens Healthcare, Erlangen, Germany). Images included T1w sequences with and without contrast agent, FLAIR (Fluid attenuated inversion recovery) sequences, T2 gradient echo sequences, diffusion-weighted imaging or diffusion-tensor imaging, whereas we calculated isotropic diffusion-weighted images and apparent diffusion coefficient (ADC) maps automatically. Tumour volumes of the contrast-enhancing tumour on pre- and early postoperative MR images using iPlannet® Cranial 3.0.1 were manually segmented by two neurosurgeons (5 and 10 years of experience) and two neuroradiologists (3 years and 6 years of experience).

In centre B, we conducted MR imaging with a 3.0 T MRI scanner (Biograph mMR, Siemens Healthcare, Erlangen, Germany). One neurosurgeon (14 years of experience) and one medical student assessed the volumes of the contrast-enhancing tumour through manual segmentation via iPlannet® Cranial 3.0.1 (iPlannet® 3.0 cranial planning software, Brainlab AG, Munich, Germany). The postoperative tumour volumes of patients who underwent biopsies were considered identical to the preoperative tumour volumes.

### Statistical evaluation

We conducted our data analysis using IBM SPSS Statistics Version 24.0 and 26.0 (SPSS Inc., IBM Corp., Armonk, NY, USA). In the descriptive data analysis, we show non-normally distributed data as median and interquartile range (IR), normally distributed variables as mean and standard deviation.

We compared the OS distributions using the Kaplan-Meier estimates (log-rank) and a Cox regression model for multivariate survival analysis. We considered differences with an error probability of less than 0.05 to be statistically significant.

## Results

### Patients and clinical data

123/968 patients (58 females/65 males) with a mean age of 67.4 ± 13.4 years; (range 21–90 years) met our inclusion criteria: surgical treatment for glioblastoma, preoperative KPSS of ≤60%, preoperative and early postoperative MRI, complete medical documentations with date of initial tumour diagnosis, date of death/last contact, age, sex, adjuvant treatment and histopathological findings. Data are shown for all patients and for the subgroups of patients with biopsy / surgery (Table 1). The median preoperative tumour volume of all patients was 33.0 ± 31.3 cm (IR 15.0–56.5cm^3)^ and the median postoperative tumour volume was 3.1 ± 23.8 cm^3^ (IR 0.2–15.0 cm^3^) postoperatively. Complete resection of contrast-enhancing tumours on postoperative MRI was achieved in 24 (19.5%) of all patients. MGMT-methylation status was available in 80 patients (65%), of whom 26 (32.5%) presented with a methylated MGMT-promotor status.

Surgical resection with intent for maximum/complete resection was performed in 75/123 patients (61%) (34/75 females and 41/75 males; mean age 64.4 ± 13.7 years (21–87 years). The median tumour volume was 35.2 cm^3^ (IR 19.7–65.3 cm^3^) preoperatively and 0.5 cm^3^ (IR 0–2.3 cm^3^) postoperatively. Complete resection of the contrast-enhancing tumour on postoperative MR imaging was seen in 24/75 patients (32%). In this group, we assessed MGMT-methylation status in 52/75 patients (69.3%). We observed methylation of MGMT in 19/52 patients (36.5%) and no methylation of MGMT in 33/52 patients (63.5%).

Fifty-eight of 75 (77.3%) patients underwent postoperative adjuvant treatment; three of 58 patients (5.1%) underwent monotherapy with temozolomide, 27/58 (46.6%) received radiation therapy only and 28/58 (48.3%) received a combined therapy according to the Stupp regime. The remaining 48 patients (38.7%) (23/48 females, 25/48 males) with a mean age of 72.1 ± 11.6 years (34–90 years) underwent biopsy for tumour histopathological diagnosis. The median tumour volume in these patients was 26.3 ± 30.9 cm^3^ (IR 8.1–51.7 cm^3^). MGMT-methylation status was available in 28 patients (58.3%) with 21/28 (75%) unmethylated MGMT promotor status. After confirming histopathological diagnosis of glioblastoma via biopsy, 8/48 (16.7%) received combined radio−/chemotherapy, 3/48 (6.3%) received chemotherapy with temozolomide only, 21/48 (43.7%) received radiotherapy alone and 16/48 (33.3%) did not receive any adjuvant therapy. To show more precisely which patients received adjuvant therapy according to the STUPP regime, we performed a correlation analysis. This showed that younger patients (*p* = 0.000) received this therapy.

Assuming that the adjuvant therapy could be started 14 days after the operation and lasted approximately 6 weeks, we could suppose the completion of the adjuvant therapy in all but nine patients on the basis of the OS. Unfortunately, the follow-up expired without documentation regarding this information.

### Karnofsky performance status scale (KPSS)

The median KPSS of the entire patient cohort was 60% (20–60%) preoperatively and 50% (0–80%) postoperatively. Seventeen patients (22.67%) who had undergone surgical tumour resection had an improved KPSS at time of discharge from the hospital, 25 patients (33.3%) remained unchanged and 33 patients (44.0%) worsened. There was no difference in the median KPSS between patients receiving surgical resection compared to patients receiving biopsy only. In the biopsied group, we recorded a median preoperative KPSS of 60% (range 40–60%) and median postoperative KPSS of 50% (range 0–70%). Patients who were treated by surgical resection showed a median preoperative KPSS of 60% (range 20–60%) and 50% (range 0–80%) postoperatively.

### Overall survival (OS)

Median OS was 5.0 months (95% CI 3.0–4.0 months) in the study population (including biopsy group and surgical resection group). Patients who received a biopsy showed a median OS of 3.0 months (95% CI 2.0–4.0 months), whereas patients who underwent surgical resection showed a median OS of 8.0 months (95% CI 3.1–12.9 months).

In order to show a possible survival advantage of the resected patients compared to the biopsied patients, we performed a Cox regression with the parameters: age, postoperative tumour volume, preoperative KPSS and biopsy. This showed no more significance for the biopsied patient group (*p* = 0.154, 95% CI 0.399–1.156).

At the time of the study, 102/123 patients (82.9%) had died, and 21/123 (17.1%) were still alive or censored for their last date of contact. In-hospital mortality was seen in 3/123 (2.4%). Two of these patients received biopsy and one surgical tumour resection.

### Univariate model

**Fig. 2:**
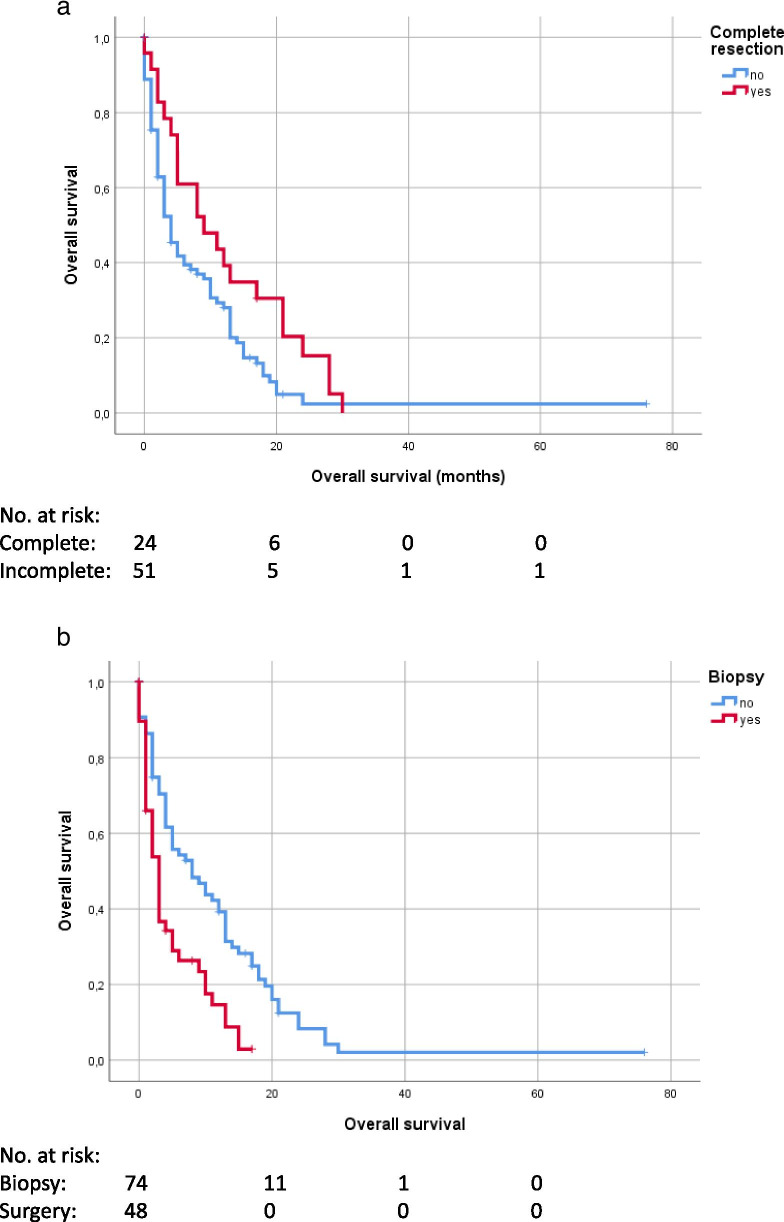
Overall survival, categorized in complete resection (A)/ biopsy (B)

**Fig. 3:**
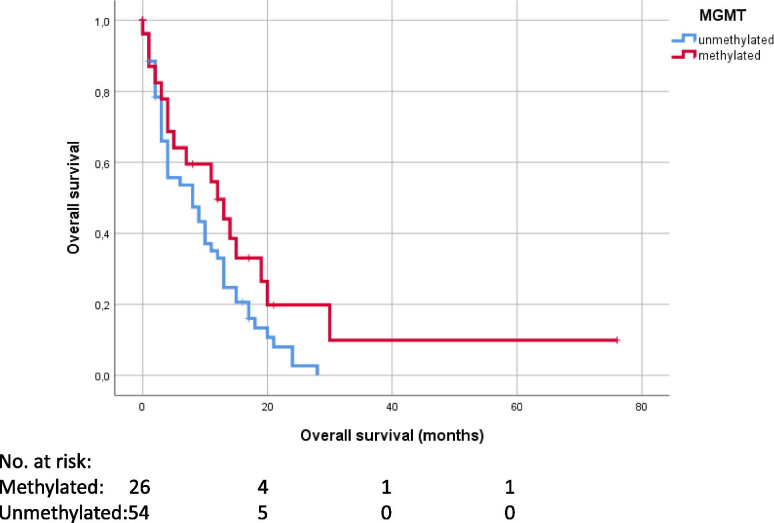
Overall survival, categorized in MGMT-methylated/MGMT-unmethylated

**Fig. 4:**
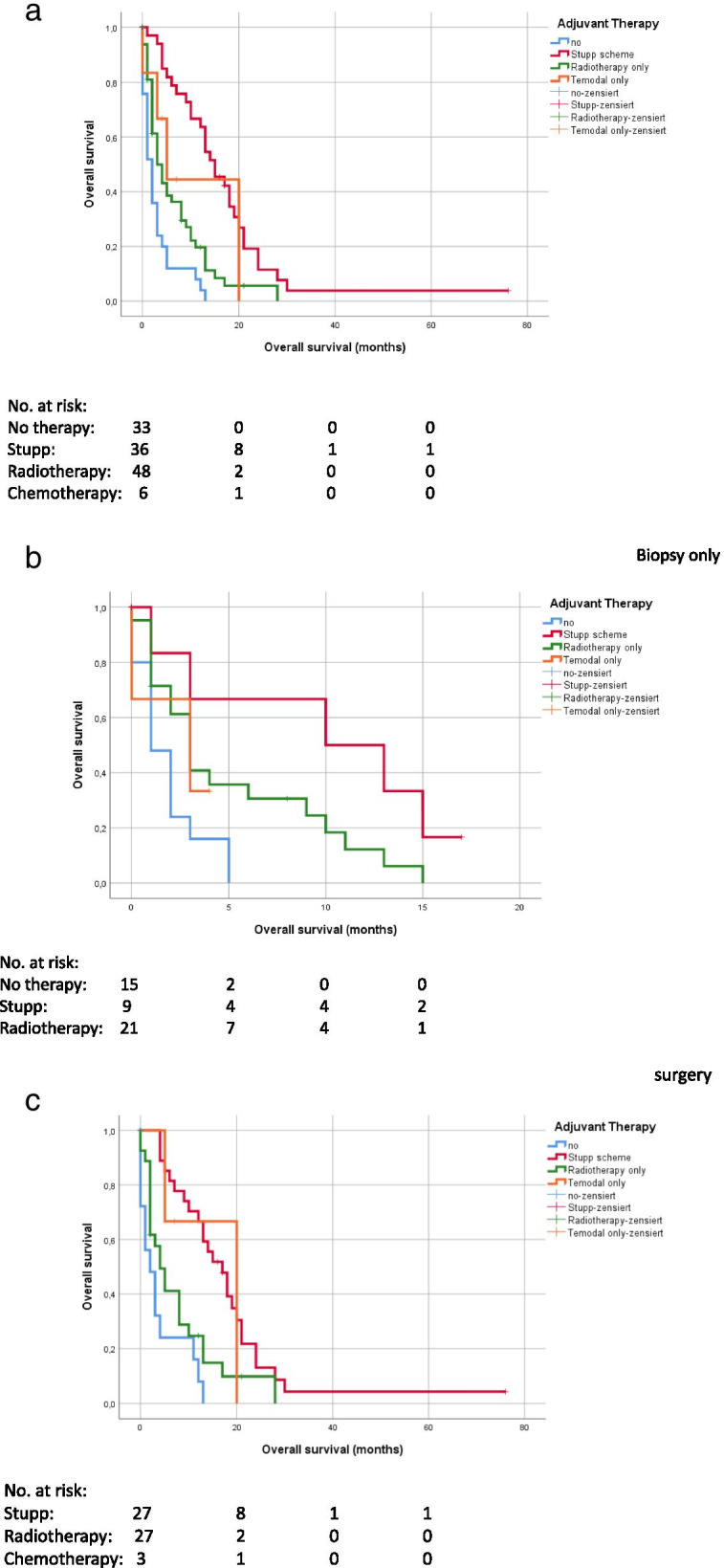
Overall survival, categorized in STUPP regime/RTX alone: A) Complete cohort B) Resection C) Biopsy

Surgical resection compared to biopsy (*p* < 0.001) and complete resection of the contrast-enhancing tumor part (*p* = 0.032) showed a significant impact on OS in the univariate analysis using Kaplan-Meier estimates. MGMT-methylation status did not show a significant impact on OS in univariate analysis (*p* = 0.071) (Figs. 2A-B and 3). Adjuvant therapy regimes also showed a significant prognostic impact, in all patients (*p* < 0.001) and in the subgroups of patients with biopsy (*p* = 0.005) and surgery (*p* < 0.001) (Fig. 4A-C).

### Multivariate model

Cox regression, including all treated patients (biopsy group and surgical resection group), showed age at the time of surgery (*p* < 0.001, HR: 1.045 [95% CI 1.022–1.068]), postoperative tumour volume (*p* = 0.02, HR: 1.016 [95% CI 1.002–1.029]) and methylation status (*p* = 0.016, HR: 0.473 [95% CI 0.257–0.871]) as statistical significant predictors of OS. Preoperative tumour volume (*p* = 0.996, HR: 1.000 [95% CI 0.992–1.009]), preoperative KPSS (*p* = 0.068, HR: 1.023 [95% CI 0.998–1.049]) and postoperative KPSS (*p* = 0.237, HR: 0.987 [95% CI 0.965–1.009]) were not significant in the multivariate analysis.

In the subgroup of patients (*n* = 75) referred for surgery only age was shown as prognostic factor in multivariate analysis: age (*p* < 0.001, HR: 1.046 [95% CI 1.022–1.072]), postoperative volume (*p* = 0.701, HR: 0.982 [0.895–1.078]), preoperative KPSS (*p* = 0.059, HR: 1.026 [0.999–1.054]).

In the subgroup of patients receiving biopsy (*n* = 48) no significant prognostic factors were shown in multivariate analysis: age (*p* = 0.086, HR: 1.028 [95% CI 0.996–1.061]), postoperative volume (*p* = 0.412, HR: 1.005 [0.993–1.017]), preoperative KPSS (*p* = 0.598, HR: 0.986 [0.936–1.039]).

## Discussion

In this cohort of GBM patients (biopsy group and surgical resection group) with a preoperative KPSS ≤60%, postoperative tumour volume, age at the time of surgery and MGMT-methylation status were significant predictors of OS in the multivariate analysis. In contrast, preoperative tumour volume and KPSS had no significant impact on OS. The subgroup analysis of patients referred for surgery only age was shown as prognostic factor in multivariate analysis, whereas no significant prognostic factors were shown in the subgroups of patients referred for biopsy. In our study population, the significant effect of postoperative tumour volume in univariate analysis correlates with tumour resection compared to the biopsied group. This could explain the lack of significance in the multivariate analysis for this group. Nevertheless, as already understood from other studies, we could also show that extent of resection is an important factor in OS in patients with glioblastoma [11–14].

In general, patients with poor preoperative KPSS usually do not receive aggressive surgical therapy. Therefore, data on these patients are very limited [15]. In our cohort, 56/123 (45.5%) showed an improved or unchanged postoperative KPSS with a median of 50%. Adjuvant treatment such as radiation therapy or chemotherapy is usually only offered to patients with a KPSS ≥70% [16, 17]. Consequently, these patients are usually considered ineligible for adjuvant oncological treatment even after tumour resection. Malakhov et al. could show that 51.2% of the patients presenting with KPSS< 60% and receiving chemoradiation had improved survival compared to RT alone [18]. However, the majority of our patient cohort (77.6%) who underwent surgical resection received adjuvant therapy. Considering the early postoperative assessment of KPSS in this study, secondary improvement is to be expected. Patients undergoing a biopsy were older (66.6%) with eloquent tumour location than patients, who were selected for surgical tumour resection (41.33%). Only 16.7% of the patients who received a biopsy underwent adjuvant treatment regimes. On the one hand, this is due to the higher age of the biopsy group. Secondly, only 7/48 of these patients showed MGMT-methylation. In accordance with the guidelines, our neuro-oncological interdisciplinary board recommends monotherapy for patients ≥75 years of age, depending on the MGMT-methylation status.

Reduced preoperative KPSS is an important prognostic factor in patients with glioblastoma [19, 20]. Age, comorbidities and neurological deficits have an impact on KPSS and, in conclusion, on OS [20–22]. Postoperative deterioration of the performance status scale is usually multifactorial, with the reasons being edema, haemorrhage, postoperative delirium, ischemic events or direct surgical lesions of eloquent brain structures [23].

In our opinion, the KPSS does not offer sufficient information about quality of life and therefore should not be overrated concerning the selection of patients undergoing surgery. For example, patients with preoperative neurological deficits such as hemiparesis due to surrounding edema might have a KPSS of 60% or below and might therefore not be selected for surgical therapy. However, as we know today, the surrounding edema will disappear a few days after surgery, and the patients are able to recover for adjuvant treatment. The KPSS should therefore be considered with care.

The decision for or against aggressive surgical therapy should be made individually by experienced neurosurgeons within the framework of an interdisciplinary neuro-oncology board.

### Limitations of the study

This study has limitations. First, the retrospective non-randomized design is the main limitation. Second, molecular status was not available for all patients in our cohort study, as the MGMT-methylation status of patients with glioblastoma is known to be one of the strongest predictors concerning survival prognosis [24, 25].

## Conclusion

GBM patients with a preoperative KPSS of ≤60% might profit from surgical reduction of tumour burden. We therefore suggest considering surgical resection even in patients with a KPSS of ≤60% after careful selection based on an interdisciplinary neuro-oncological board decision and counselling of patients and their relatives.

## Data Availability

The datasets used and/or analysed during the current study are available from the corresponding author on reasonable request.

## Abbreviations

KPSS: Karnofsky Performance Status Scale
OS: Overall survival
GBM: Glioblastoma
MGMT: O6-methylguanin-DNA-methyltransferase
EOR: Extent of resection
HR: Hazard ratio
IR: Interquartile range
GTR: Gross tumor resection
FLAIR: Fluid attenuated inversion recovery
ADC: Apparent diffusion coefficient
